# Impact of Intrapartum Oral Azithromycin on the Acquired Macrolide Resistome of Infants’ Nasopharynx: A Randomized Controlled Trial

**DOI:** 10.1093/cid/ciaa609

**Published:** 2020-05-23

**Authors:** Abdoulie Bojang, Sarah L Baines, Bully Camara, Romain Guerillot, Liam Donovan, Raquel Sánchez Marqués, Ousman Secka, Umberto D’Alessandro, Christian Bottomley, Benjamin P Howden, Anna Roca

**Affiliations:** 1 Medical Research Council Unit, The Gambia, at the London School of Hygiene and Tropical Medicine, Fajara, The Gambia; 2 Department of Microbiology and Immunology, The University of Melbourne at The Peter Doherty Institute for Infection and Immunity, Melbourne, Victoria, Australia; 3 Tropical Epidemiology Group, London School of Hygiene and Tropical Medicine, United Kingdom; 4 Microbiological Diagnostic Unit Public Health Laboratory, Department of Microbiology and Immunology, The University of Melbourne at The Peter Doherty Institute for Infection and Immunity, Melbourne, Victoria, Australia

**Keywords:** azithromycin, *erm*C, *msr(A)*, NPS, The Gambia

## Abstract

In a post hoc analysis of samples from an intrapartum azithromycin randomized clinical trial, we found that children whose mothers had been treated with the drug had higher prevalence of macrolide-resistance genes *msr(A)* and *ermC* at 28 days but not at 12 months. The 2 genes were positively associated in the nasopharynx.

**Clinical Trials Registration:**

NCT1800942.

Prophylactic use of azithromycin (AZI) has been investigated in low- and middle-income countries as an intervention to decrease infant mortality [1].

In a recent double-blinded, placebo-controlled trial conducted in The Gambia (PregnAnZI), a single dose of oral AZI (2 g) was administered during labor to assess the impact on bacterial colonization of gram-positive bacterial species (*Staphylococcus aureus*, *Streptococcus pneumoniae*, and group B *Streptococcus*) in both mothers and their offspring. The trial showed that this intervention reduced bacterial colonization in women and infants during the 4 weeks after birth but increased the prevalence of AZI-resistant *S. aureus*, although in a follow-up study of the trial participants it was observed that carriage of AZI-resistant *S. aureus* strains had waned by the age of 12 months [2].

Molecular analysis of the AZI-resistant *S. aureus* strains isolated in the PregnAnZI trial revealed that the predominant genetic determinants responsible for macrolide resistance in this population were macrolide and streptogramin A resistance msr(A) and erythromycin ribosomal methylase C (ermC) [3]. Both these genes are carried by mobile genetic elements; hence, horizontal spread between bacteria colonizing the same ecological niche could take place through transformation, conjugation, or transfection [4–6]. In fact, *msr(A)* and *ermC* are also present in other bacteria that colonize the nasopharynx such as *Staphylococcus* species and *Enterococcus* species [7–11]. Consequently, screening a single bacterium (ie, *S. aureus*) may underestimate the true prevalence for resistance. The aim of this post hoc study was to evaluate the effect of intrapartum AZI on the prevalence of the macrolide-resistance genes [*msr(A)* and *ermC*] in the nasopharynx at different time points during infancy.

## METHODS

The PregnAnZI trial was a phase III, double-blind, placebo-controlled trial in which 829 pregnant women attending the maternity ward were randomized to receive either a single oral dose (2 g) of intrapartum AZI or placebo (ratio of 1:1). The study protocol has been described elsewhere [12]. After completion of the trial, nasopharyngeal swab (NPS) were collected in a follow-up survey done when the children were between 11 and 13 months of age. Ethical approval was obtained for both the main trial and the 12-month survey. Women had signed consent during antenatal visits and signed another consent for the infant’s follow-up visit.

Only children from whom NPS were collected at birth (day 0), day 28, and at 12 months were eligible for inclusion in this post hoc study. The study was conducted using 936 samples from 312 children (n = 155 AZI arm and n = 157 placebo arm) who were selected at random from among the eligible children (n = 426).

Genomic DNA was extracted directly from NPS using the QIAamp DNA Mini Kit (Qiagen, United Kingdom) protocol with some modifications. The DNA was eluted in 100-µL volume and stored at −20ºC.

The primers for the amplification of the macrolide resistance genes [*msr(A)_*F ATCCAATCATTGCACAAAATCTAACATT, *msr(A)_*R TAAATAGCTTCAAGTAAAGTTGTCTTACC and *ermC*_F CTTGTTGATCACGATAATTTCCAAG, *ermC*_R TTGTATTCTTTGTTAACCCATTTCATAAC] were designed using Primer 3 and synthesized by Metabion, Germany. The cycling conditions included an initial denaturation at 98ºC for 30 seconds, 35 cycles of denaturation at 98ºC for 5 seconds, annealing at 52ºC for 5 seconds, and extension at 72ºC for 10 seconds, with a final extension at 72ºC for 1 minute. Fully sequenced *S. aureus* carrying either the *ermC* or *msr(A)* genes were used as positive controls.

Polymerase chain reaction (PCR) analyses were performed alongside DNA extract from a pure *S. aureus* isolate known to carry either the *ermC* or *msr(A)* gene (positive controls) [3]. The PCR products were analyzed using QIAxcel advanced Screen gel 1.5.0 (Qiagen) [13] with a tolerance rate of  ±15% of the expected band size [*msr(A)* 145 bp and *ermC* 398 bp]. Samples were categorized as being either positive or negative for each resistance gene.

Pearson’s χ ^2^ test was used to compare the prevalence of macrolide-resistance genes between arms at birth, day 28, and at 12 months. The χ ^2^ test was also used to test for an association between the resistance genes at day 28. This analysis was further stratified by trial arm, and a Mantel-Haenszel test for interaction was done to test whether the strength of association varied between arms. A *P* value ≤ .05 was used as the cutoff for statistical significance. All analyses were carried out using Stata version 12.1 software (StataCorp).

## RESULTS

### Study Population and Samples

Baseline characteristics of the study mothers (maternal age at delivery, fundal height, mode of delivery, ethnicity, and season of birth) and their infants (sex, birth weight, and Apgar score) selected for this post hoc study were comparable between the AZI and placebo arms (see Supplementary Table 1).

### Prevalence of Macrolide-resistance [*Msr(A)* and *ErmC*] Genes in Infants

The prevalence of *msr(A)* at birth was similar between trial arms (25.2% vs 25.5% in the AZI and placebo arms, respectively). At day 28, the prevalence was higher among children in the AZI arm (60.7% vs 29.9%; odds ratio [OR], 3.61; 95% confidence interval [CI], 2.20–5.93]), but the difference had waned by the age of 12 months (see Table 1).

**Table 1.** Prevalence of *msr(A)* and *ermC* Genes by Trial Arm

Gene	Day 0				Day 28				Year 1																																														
	AZI (n = 155), n (%)	Placebo (n = 157), n (%)	OR (95% CI)	*P*	AZI (n = 155), n (%)	Placebo (n = 157), n (%)	OR (95% CI)	*P*	AZI (n = 155), n (%)	Placebo (n = 157), n (%)	OR (95% CI)	*P*
*msr(A)*												
Yes	39 (25.2)	40 (25.5)			94 (60.7)	47 (29.9)			27 (17.4)	29 (18.5)		
No	116 (74.8)	117 (74.5)	.98 (.57–1.69)	.949	61 (39.3)	110 (70.1)	3.61 (2.20–5.93)	<.001	128 (82.6)	128 (81.5)	.93 (.50–1.73)	.809
*erm*C												
Yes	47 (30.3)	55 (35.0)			99 (63.9)	72 (45.9)			80 (51.6)	68 (43.3)		
No	108 (69.7)	102 (65.0)	.81 (.49–1.33)	.375	56 (36.1)	85 (54.1)	2.09 (1.29–3.37)	.001	75 (48.4)	89 (56.7)	1.40 (.87–2.24)	.142

Abbreviations: AZI, azithromycin; CI, confidence interval; OR, odd ratio.

The prevalence of *ermC* was also similar between trial arms at birth (30% vs 35.0% in the AZI and placebo arms, respectively). As with *msr(A)*, the gene was more common among children in the AZI arm at day 28 (63.9% vs 45.9%; OR, 2.09; 95% CI, 1.29–3.37) and the difference was no longer significant by the age of 12 months (see Table 1).

### Association of *msr(A)* and *ermC* Genes at Day 28

At day 28, there was a positive association between the *ermC* and *msr(A)* genes (OR, 2.31; 95% CI, 1.42–3.76). Although the association appeared to be stronger in samples from the placebo arm (OR, 2.86; 95% CI, 1.33–6.20) than in the AZI arm (OR, 1.41; 95% CI, .68–2.90), there was no evidence of an interaction by study arm (*P* = .162).

## DISCUSSION

Our analysis shows that although intrapartum AZI increases carriage of macrolide-resistance genes [*msr(A)* and *ermC*] in the first month of life, and the increase is not sustained by 1 year. This short-term increase in genetic mediators of resistance mirrors the pattern of phenotypic resistance for *S. aureus* that has previously been reported [2, 14].

Data on *msr(A)* and *ermC* gene prevalence following prophylactic AZI are scarce. In The Gambia, cases of AZI-resistant *S. aureus* from all age groups in the community following mass drug administration with AZI were attributed to the presence of either *msr* or *erm(C)* genes [15]. A study conducted in Australia and New Zealand found a nonsignificant increase in carriage of *msr(A)* in patients with non–cystic fibrosis bronchiectasis on long-term erythromycin, a sister macrolide to AZI [16]. Another study conducted in children under 5 years in Niger found that children who received twice-a-year AZI had an approximately 30% higher prevalence of the streptococcal macrolide-resistance determinant *mefA/E*, an equivalent of the staphylococcal *msr(A)* gene, than did children who received AZI once per year [17]. Although formal comparisons between the aforementioned trial and our trial are difficult because of the different designs (community seasonal prophylaxis vs single-dose intrapartum administration), it appears that *mefA/E* persisted longer within the population than *msr(A)* or *ermC*. We did not include *mefA/E* in our study as the gene is predominantly associated with *Streptococci*, and in our trial AZI resistance following intrapartum oral intervention was only significantly increased for *S. aureus* and not *S. pneumoniae* [2, 14].

The increase in *ermC* or *msr(A)* prevalence observed at day 28 is associated with increased macrolide resistance in the study population. The presence of the *msrA* gene is indicative of phenotypic resistance to 14-membered (clarithromycin, dirithromycin, and erythromycin) or 15-membered (AZI) macrolides as well as streptogramin A but sensitive to 16-membered ring macrolides. On the other hand, the presence of the *ermC* gene is also associated with an even wider scope of phenotypic resistance, including resistance to clindamycin and streptogramin B, depending on whether the presence of the gene results in an inducible or constitutive phenotype [18]. All *S. aureus* isolates carrying *ermC* from our previous molecular and phenotypic analysis showed constitutive resistance [2]. Like the *msr(A)* gene, the prevalence of *ermC* at 12 months was nonsignificantly higher in the AZI arm than in the placebo arm. However, additional studies should determine whether the *ermC* gene, beyond being as prevalent as *msr(A)*, is also more persistent as its prevalence in the AZI arm was 51.6% compared with 17.4% for the *msr(A)* gene at 12 months.

In our previous molecular analysis of *S. aureus* isolates, we observed a negative association between *ermC* and *msr(A)* [3]. In contrast, the current analysis identified a positive association between the 2 genes. Taken together, these results suggest that macrolide-resistance genes tend to co-occur in the same sample but are carried by different bacterial isolates or species. *Staphylococcus epidermidis*, for example, has been reported to carry both *ermC* and *msr(A)* genes [11].

Key limitations of the study include that it was not possible to (1) determine for how long beyond 28 days AZI resistance was maintained, (2) relate the prevalence of the macrolide-resistance genes to changes in microbiome diversity, and (3) ascertain whether the *msr(A)* and *ermC* genes detected by PCR were functional.

In conclusion, screening for macrolide-resistance genes following intrapartum AZI exposure revealed increased prevalence of both *msr(A)* and *ermC* during the neonatal period but not at 12 months of age. Genetic surveillance should be used to complement conventional antimicrobial resistance surveillance in the event prophylactic AZI interventions are rolled out in The Gambia or elsewhere.

## Supplementary Data

Supplementary materials are available at *Clinical Infectious Diseases* online. Consisting of data provided by the authors to benefit the reader, the posted materials are not copyedited and are the sole responsibility of the authors, so questions or comments should be addressed to the corresponding author.

ciaa609_suppl_Supplemental-Data

## Notes

***Author contributions.*** The project was conceived by A. R., B. P. H., and A. B. B. C. collected samples from the main trial. A. B. performed the bulk of the experimental work, with additional work performed by S. L. B., R. S. M., and O. S. A. B., L. D., and S. L. B. designed the primers, with additional input from R. G. C. B. assisted with statistical analyses. A. B. and A. R. drafted the manuscript, with input from all co-authors. All authors approved the final version of the manuscript.

***Acknowledgments***. The authors are grateful to the field team for assisting with sample collection and to the study participants for agreeing to take part in the study. Special thanks to Kaddy Saho for helping with the laboratory analysis and Abdoulie Jabang for technical support in the use of the QIAxcel.

***Financial support***. This work was jointly supported by the Medical Research Council (MRC) Unit, The Gambia, at the London School of Hygiene and Tropical Medicine PhD fellowship awarded to A. B. The main trial was jointly funded by the UK MRC and the UK Department for International Development (DFID) under the MRC/DFID Concordat agreement (reference number MR/J010391/1) and is also part of the EDCTP2 program supported by the European Union.

***Potential conflicts of interest.*** The authors: No reported conflicts of interest. All authors have submitted the ICMJE Form for Disclosure of Potential Conflicts of Interest.
