# Supplementary material for: Impact of Intrapartum Oral Azithromycin on the Acquired Macrolide Resistome of Infants’ Nasopharynx: A Randomized Controlled Trial
Source: Clin Infect Dis. 2020 May 23;71(12):3222–5. doi: 10.1093/cid/ciaa609 (PMC7819521; doi:10.1093/cid/ciaa609)
Supplement: ciaa609_suppl_Supplemental-Data [file ciaa609_suppl_supplemental-data.docx]

**SUPPLEMENTARY DATA**

| Supplementary S1. Baseline characteristics of study mothers and babies | | | | |
| --- | --- | --- | --- | --- |
| Characteristics | | **AZI n= 155** | **Placebo n= 157** | **p value** |
| Mother information | |  |  |  |
| Maternal age(years) at delivery, mean (SD) | | 26.7(5.4)^a^ | 26.9(5.0) |  |
| Fundal height(cm), mean (SD)^b^ | | 35.8(2.6) | 35.8(3.3) |  |
| Mode of delivery | |  |  |  |
| Vaginal | | 155(100) | 157(100) |  |
| Caesarean | | 0 | 0 |  |
| Ethnicity |  | |  | 0.405 |
| Mandinka | | 68(43.9) | 68(43.3) |  |
| Jola | | 30(19.4) | 26(16.6) |  |
| Wollof | | 21(13.6) | 14(8.9) |  |
| Fula | | 18(11.6) | 27(17.2) |  |
| Sarahule | | 7(4.5) | 5(3.2) |  |
| Others | | 11(7.1) | 17(10.8) |  |
| Season of birth^c^ | |  |  |  |
| Dry | | 114(73.6) | 118(75.2) | 0.745 |
| Wet | | 41(26.5) | 39(24.8) |  |
| Child information at birth up to day 28 | |  |  |  |
| Birthweight(kg), mean (SD) | | 3.13(0.5) | 3.04(0.4) |  |
| Apgar score^d^ | |  |  |  |
| 9 – 10 | | 142(92.2) | 147(93.6) |  |
| 7 - 8 | | 12(7.8) | 9(5.7) |  |
| ≤6 | | 0 | 1(0.7) |  |
| Twins | | 1(0.7) | 4(2.6) | 0.181 |
| Singleton | | 154(99.3) | 153(97.4) |  |
| Sex^e^ | |  |  |  |
| Female | | 83(53.9) | 73(47.4) | 0.254 |
| Male | | 71(46.1) | 81(52.6) |  |

Data are presented as No. (%) unless otherwise indicated.

Abbreviation: SD, standard deviation.

^a^one missing data item in the azithromycin arm. ^b^6 missing data (2 in the azithromycin arm and 4 in the placebo arm). ^c^wet season = June to October, dry season = November to May ^d^1 missing data in the AZI arm ^e^4 missing data (1 in AZI and 3 in placebo arm)
